# Comparison of angiogenic potential in vitrified vs. slow frozen human ovarian tissue

**DOI:** 10.1038/s41598-023-39920-x

**Published:** 2023-08-09

**Authors:** Andreas Schallmoser, Rebekka Einenkel, Cara Färber, Vanessa Hüren, Norah Emrich, Julia John, Nicole Sänger

**Affiliations:** Department of Gynecological Endocrinology and Reproductive Medicine, University Hospital of Bonn, Venusberg Campus 1, 53127 Bonn, Germany

**Keywords:** Biological techniques, Molecular biology, Medical research, Molecular medicine, Oncology

## Abstract

Vitrification of ovarian tissue is a promising alternative approach to the traditional slow freezing method. Few empirical investigations have been conducted to determine the angiogenic profiles of these two freezing methods. In this study we aimed to answer the question whether one of the cryopreservation methods should be preferred based on the secretion of angiogenic factors. Tissue culture with reduced oxygen (5%) was conducted for 48 h with samples of fresh, slow frozen/thawed and vitrified/rapid warmed ovarian cortex tissue from 20 patients. From each patient, tissue was used in all three treatment groups. Tissue culture supernatants were determined regarding cytokine expression profiles of angiogenin, angiopoietin-2, epidermal growth factor, basic fibroblast growth factor, heparin binding epidermal growth factor, hepatocyte growth factor, Leptin, Platelet-derived growth factor B, placental growth factor and vascular endothelial growth factor A via fluoroimmunoassay. Apoptotic changes were assessed by TUNEL staining of cryosections and supplemented by hematoxylin and eosin and proliferating cell nuclear antigen staining. Comparing the angiogenic expression profiles of vitrified/rapid warmed tissue with slow frozen/thawed tissue samples, no significant differences were observed. Detection of apoptotic DNA fragmentation via TUNEL indicated minor apoptotic profiles that were not significantly different comparing both cryopreservation methods. Vitrification of ovarian cortical tissue does not appear to impact negatively on the expression profile of angiogenic factors and may be regarded as an effective alternative approach to the traditional slow freezing method.

## Introduction

Ovarian tissue cryopreservation (OTC) is an integral part of female fertility preservation. While it expands the range of fertility preservation measures besides oocyte cryopreservation in post-menarche women^1–3^, it is the only option for fertility preservation in pre-menarche girls. Increasing success rates after transplantation of ovarian tissue could be seen in the past decade, substantiating that OTC is a safe alternative method of fertility preservation^4–6^.

The majority of centers performing OTC use the slow freezing method while the optimal protocol regarding vitrification of ovarian tissue has yet to be determined^7^.

Suzuki et al. 2015 and Silver et al. 2018 reported 4 successful deliveries originating from vitrified/rapid warmed transplanted ovarian tissue^8, 9^. Thus, vitrification of ovarian tissue is an alternative approach besides slow freezing in a clinical setting.

Ice formation during the slow freezing procedure potentially damages tissue components. Nonetheless, the effectiveness of this method has been proved in many species^10^. Optimized parameters of slow freezing conditions, including cooling and warming rates beside the quantitative and qualitative composition of the used cryoprotective agents (CPA) safeguard the tissue against cryodamage. With vitrification, a glassy state of aggregation is formed by increasing viscosity^11^. With vitrification no ice crystal formation is observed^11, 12^. In a meta-analysis of 14 human studies comparing vitrification with slow freezing, Shi and colleagues observed no difference regarding follicular viability while vitrification was associated with decreased DNA-damage^13^. Reports of expression profiles of angiogenic factors after hypoxic tissue culture of cryopreserved thawed ovarian tissue are limited. In mice^14, 15^, significant differences between slow frozen and vitrified samples were observed, while other groups report partially conflicting results in humans^16–18^, baboons^19^ and mice^20^. This may be due to the use of various tissue vitrification protocols, different quantitative and qualitative composition of cryoprotective agents (CPA) and carrier systems. The studies presented thus far provide important insights substantiating that further work is required to establish an optimal vitrification protocol, that gives superior results in terms of follicular survival and tissue integrity in a clinical routine setting.

In our recent study, we describe a high throughput vitrification protocol suitable for clinical routine without detecting significant differences regarding follicular viability in comparison to the slow freezing standard protocol^7^. In this prospective study, we analysed the secretion of 10 angiogenic factors after 48 h of hypoxic tissue culture of human ovarian tissue. Thawed slow frozen samples were compared with rapid warmed vitrified tissue. Since the secretion of angiogenic factors is regulated by stress responses, analysis was complemented by tissue sections stained for apoptosis (TUNEL).

## Methods

### Ethics

The study was approved by the ethics committee of University Hospital Bonn (007/09). Written, informed consent was obtained individually from each patient enabling the use of 10% of the ovarian tissue for patient related research and quality control. We confirm that all research was performed in accordance with declaration of Helsinki.

### Statistics

Data Analysis was performed with SPSS version 25 (IBM) and GraphPad Prism 9 statistical software. To test for differences between groups non-parametric ANOVA (Friedman's test) with post hoc Dunn-Bonferroni calculations were used.

### Ovarian tissue

Ovarian tissue was obtained from 20 patients aged 10–35 (Ø 27.9 y) at the time of cryopreservation for fertility protective measures.

### Study design

**Figure 1:**
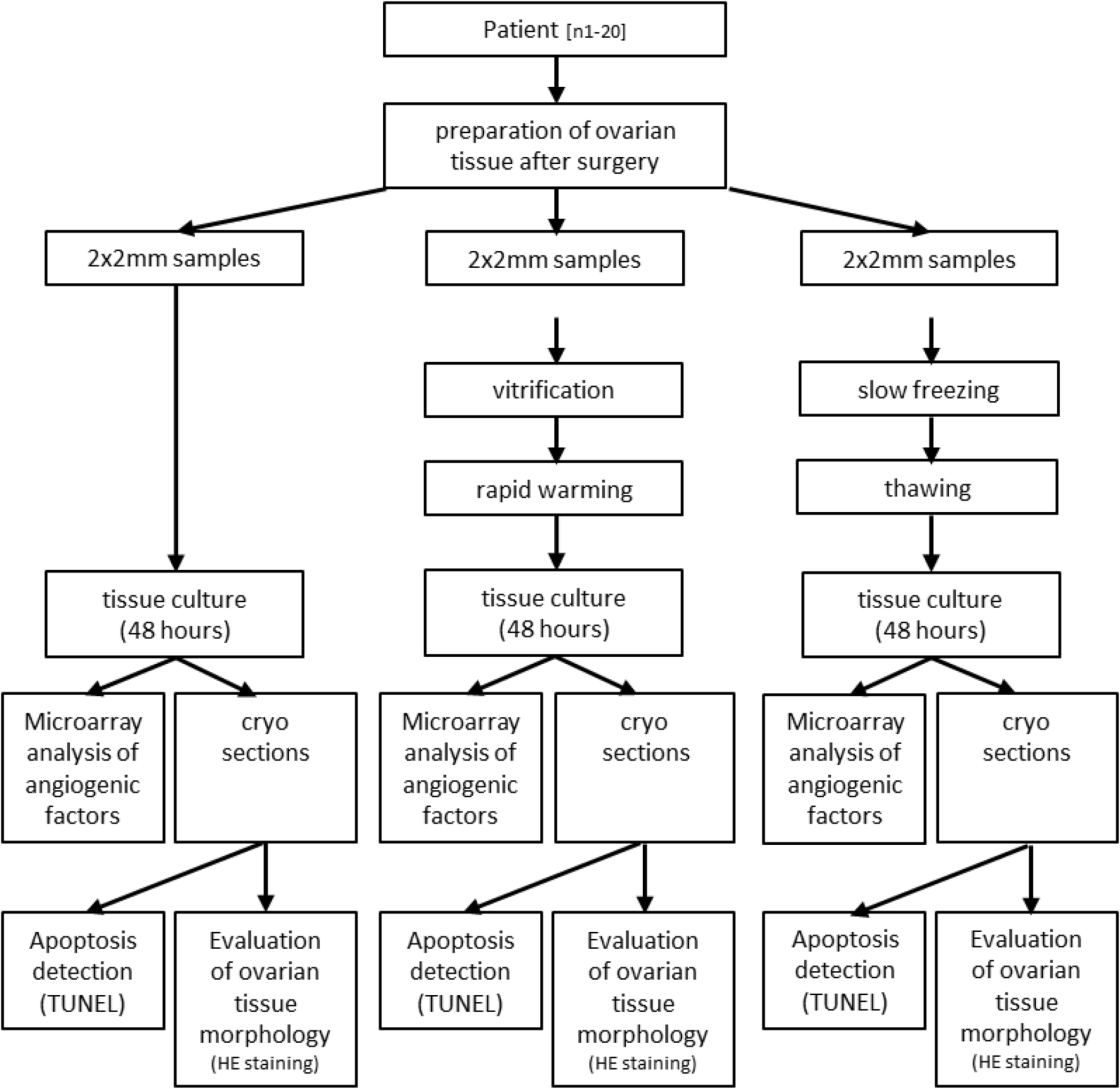
Experimental design of the study.

Ovarian cortex tissue from each of 20 patients was split into 3 groups (fresh, slow frozen/thawed and vitrified/rapid warmed) for tissue culture, angiogenic profiling and apoptosis assay as indicated in Fig. 1.

### Surgical retrieval, transportation and tissue preparation prior to cryopreservation

Outpatient surgery was performed laparoscopically with sharp scissors, without applying electrocoagulation before gonadotoxic treatment of cancer. 30–50% of one ovary was removed, depending on the extent of expected gonadal toxicity. A small sample of the ovarian tissue was sent to the histopathological department to exclude malignant cells. Tissue was transferred to a sterile 30 ml tube with precooled (2–8 °C) custodiol (Dr. Köhler, Bensheim, Germany) perfusion solution. Transportation was conducted by commercial providers in an isolated box containing three precooled pairs of thermal packs, capable of maintaining a temperature of 2–8 °C for 22 h. Tissue transport was conducted via overnight transportation, as cryobanks in Germany are centralised and therefore not available at every hospital. After arrival, tissue was processed on a coldplate at 4 °C in prechilled (2–8 °C) custodiol (Dr. Köhler, Bensheim, Germany). After removal of the medulla, cortex was customized to stripes sized 10 × 5 × 1 mm.

**Figure 2:**
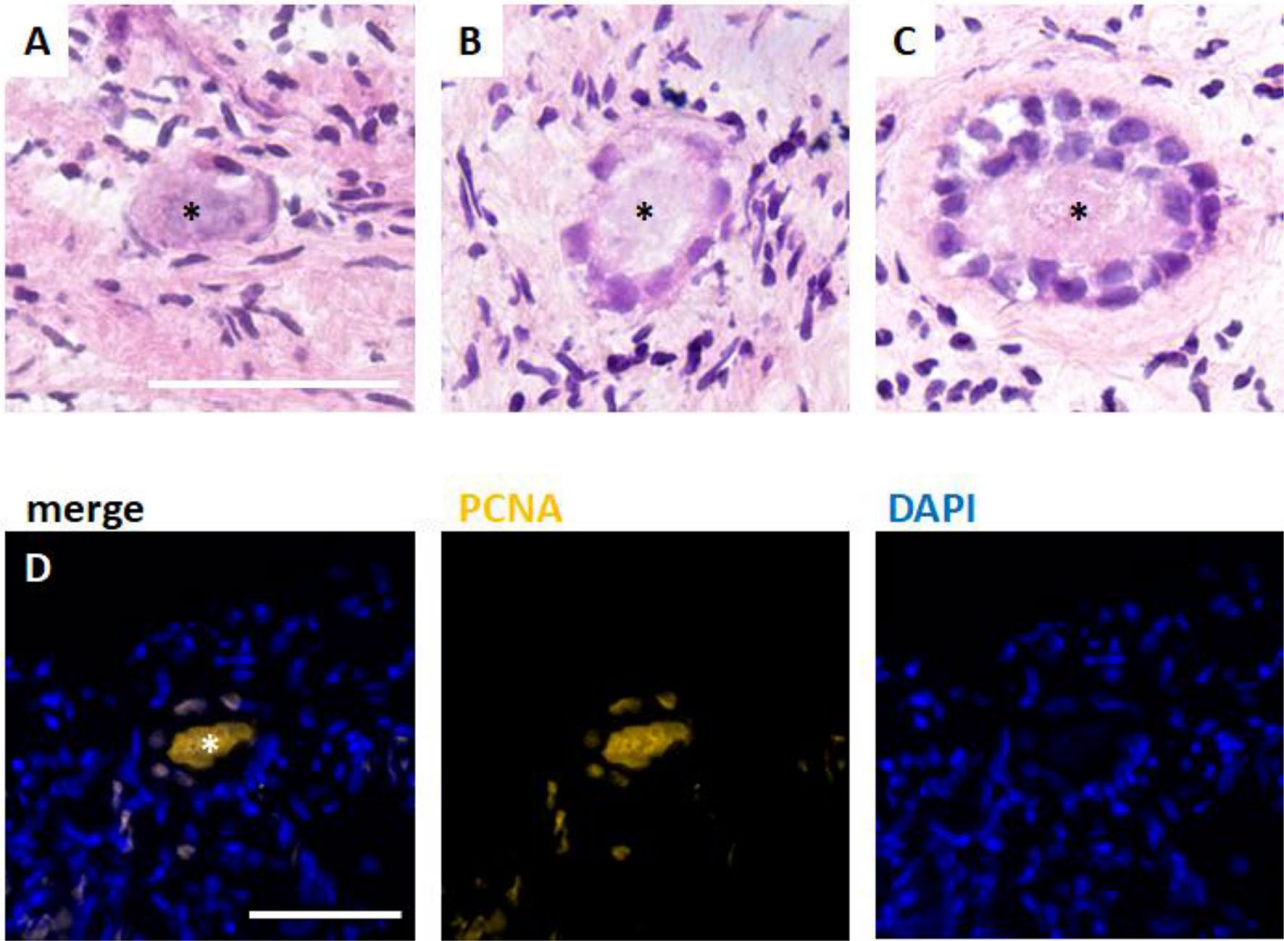
Histology of follicles in human ovarian tissue after in vitro culture without cryopreservation or after either vitrification/rapid warming or slow freezing/thawing. Ovarian tissue was stained by either (**A**–**C**) HE staining or (**D**) immunohistofluorescence after hypoxic in vitro culture. Representative images are shown. Primordial (**A**), primary (**B**) and secondary follicles (**C**) defined by the appearence of the granulosa cells were observed in all treatment groups. The follicle marker PCNA (proliferating cell nuclear antigen, yellow) was stained and counterstained with DAPI (blue) to confirm viability. Scale bars mark the length of 50 µm. *Asterisks mark oocytes.

Samples of 20 patients with written consent and sufficient sample size were included (see Table 1). Tissue pieces obtained by 2 mm diameter tissue punch (pfm medical, Cologne, Germany) were used for this study. Tissue was used freshly, or cryopreserved as described below, stored in liquid nitrogen and warmed/thawed prior to tissue culture (see Fig. 1). Samples from each patient were included in all three groups to minimize inter-individual variability potentially affecting the outcome of this study (Fig. 2).

**Table 1:** Patient characteristics.

Patient number	Indications	Age [years] at time of cryopreservation	2 mm diameter samples* [n] cultured fresh, after slow freezing/thawing, after vitrification/rapid warming	Overnight transport [hours]	Temperature at arrival on site [°C]
20	Acute lymphocytic leukemia (ALL), breast cancer (7), T-cell lymphoma, hodgkin lymphoma (3), neoplasms of the appendix, desmoid fibromatosis, melanoma (2), aplastic anaemia, ovarian cancer (2), borderline ovarian tumour	27.9 [average]	120 (40/40/40)	19.7 [average]	6.2 [average]
		30.5 [median]		20 [median]	6 [median]
		10 [min]		16 [min]	4 [min]
		35 [max]	*2 per patient, per treatment group	21.9 [max]	8 [max]
		24.75 [Q1]		19 [Q1]	5 [Q1]
		30.5 [Q2]		20 [Q2]	6 [Q2]
		5.75 [IQR]		1 [IQR]	1 [IQR]

Tissue was surgically retrieved, prepared and cryopreserved in the time period from 21.12.2021 to 21.10.2022.

### Vitrification and rapid warming

Preparation of vitrification and rapid warming solutions were performed according to Suzuki and colleagues with modifications described elsewhere^7, 8^. In brief, tissue was equilibrated in different solutions with varying concentrations of 10%, 20% and finally 35% ethylene glycol (Merck, Darmstadt, Germany) in GMOPS+ (Vitrolife, Gothenburg, Sweden) supplemented with 10% SSS (Serum substitute supplement, Fujifilm Irvine scientific, Santa Ana, USA) for 5 min each. The solution of 35% ethylene glycol was additionally supplemented with 5% polyvinylpyrrolidon [PVP] (Merck, Darmstadt, Germany) and 0.5 mol/L sucrose (Merck, Darmstadt, Germany). Subsequently, surplus solution was removed with sterile cellulose material and the tissue was fast loaded on customized metal meshes prior to immediate vertical immersion in liquid nitrogen.

Samples were rapid warmed submerging cortex strips in warming solution with decreasing sucrose gradients supplemented with 10% SSS in GMOPS+. Tissue was submerged in 0.8 mol/L sucrose for 1 min at 37 °C and equilibrated in 0.4 mol/L sucrose for 3 min. Tissue was then washed in GMOPS+ supplemented with 10% SSS for 5 min twice.

### Slow freezing and thawing

Cortex samples were slow frozen and thawed according to published protocols with modifications^21–26^. In brief, tissue was equilibrated in L-15 Leibovitz’s medium (Gibco Life technologies, NY, U.S.A.) supplemented with 11% human serum albumin [HSA] (Irvine Scientific, Santa Ana, USA), 10% dimethyl sulfoxide [DMSO] (CryoSure DMSO, WAK Chemie, Steinbach, Germany) for 40 min prior to slow freezing procedure at a cooling rate of − 2 °C per min. After completion of the seeding procedure, a cooling rate of − 0.3 °C per min was conducted to − 40 °C and at − 10 °C per min to − 140 °C. Freezing was conducted with IceCube 14S (Sy-Lab, Purkersdorf, Austria). After storage in liquid nitrogen, samples were thawed at room temperature for 30 s and submerged in a water bath for 130 s at 37.2 °C prior to transfer to a decreasing sucrose gradient (0.75, 0.375, 0.187 M) in CTS DPBS (Life technologies, Carlsbad, CA, USA) supplemented with 11% HSA (Irvine Scientific, Santa Ana, USA) for 15 min each. This was followed by 2 washing intervals in CTS DPBS supplemented with 11% HSA for 15 min at first and subsequent 5 min.

### Ovarian tissue culture

Groups of 2 × 2 mm diameter tissue pieces per patient were cultured in 200 µl of a bicarbonate buffered medium containing hyaluronan and human serum albumin (GTL Vitrolife, Germany), supplemented with 10% SSS (Fujifilm Irvine scientific, Santa Ana, USA) under oil (GM501 Mineral Oil, Gynemed, Lensahn). Culture was performed in 60 mm center well dishes (Oosafe, Sparmed, Denmark) at 37.1 °C and 6.5% CO_2_, to maintain a stable pH between 7.2 and 7.4 under hypoxic conditions [5% O_2_]. For incubation, G185 flatbed incubators (K-Systems, Cooper surgical, Berlin, Germany) were used.

### Angiogenesis array

In order to assess the secretion of angiogenic factors by the cultured ovarian cortical tissue, the Human Angiogenesis Array (RayBiotech Life, Peachtree Corners, GA, USA) was used. Thus, the relative secretion of angiogenin, angiopoietin-2, EGF, bFGF, HB-EGF, HGF, Leptin, PDGF-BB, PLGF and VEGF were measured. Following the manufacturer’s protocol, the array was thawed and dried for 2 h before usage. The array was then incubated with 100 µL Sample Diluent in each well for 30 min. Tissue culture supernatant samples 1:2 diluted in Sample Diluent were then incubated on the array overnight at 4 °C. Slide was washed 5 times for 5 min in Wash Buffer I and 2 times for 5 min in Wash Buffer II. Each well was incubated with 80 µL of the Detection Antibody Cocktail for 1 h and washed as described before. Cy3 Equivalent Dye-Conjugated Streptavidin was added and incubated for 1 h in the dark. To wash off, the slide was washed 5 times for 5 min in Wash Buffer I, disassembled and washed in Wash Buffer I for 15 min and in Wash Buffer II for 5 min. Finally, the slide was rinsed with ddH2O. The array was analysed using the InnoScan 710 and the MAPIX Software (Innopsys; Carbonne, France) at 532 nm. The mean signals of quadruplicates were compared. Per array one negative control (unconditioned medium only) was measured.

### Cryosections

After culture, tissue was fixed in 3.7% formalin (Carl Roth, Karlsruhe, Germany) in PBS (PAN Biotech, Aidenbach, Germany) at 4 °C overnight. The fixed tissue was then incubated in 15% and 30% sucrose in PBS for 10 min each. Afterwards, it was embedded in CryoGlue (SLEE; Nieder-Olm, Germany), frozen in liquid nitrogen and stored at − 20 °C. Tissue sections of 5 µm thickness were conducted using the MEV cryostat (SLEE; Nieder-Olm, Germany). Sections were dried onto the slide at 37 °C for 1 h.

### HE staining

Cryosections were stained with hematoxylin and eosin (HE). The staining was conducted using the H&E fast staining kit (Carl Roth, Karlsruhe, Germany). After washing in water for 10 s, the slides were incubated with the hematoxylin solution for 6 min. The slides were then rinsed in tap water and incubated in hydrochloric acid for 10 s. Blueing was conducted in tap water for 6 min. The slides were incubated in eosin for 1 min. The excess was washed off. For mounting, sections were dehydrated in 90% ethanol (Carl Roth, Karlsruhe, Germany) and absolute ethanol twice for 5 min. It was cleared in xylene twice for 5 min. Finally, cryosections were mounted in Eukitt (Sigma-Aldrich, St. Louis, MI, USA). Images were taken using the Eclipse Ti2 microscope (Nikon; Minato, Japan).

### Immunohistofluorescence staining

PCNA (proliferating cell nuclear antigen) is expressed in proliferating cells, but also in oocytes. Since there is a strong expression in follicles, PCNA is used as follicle marker in human ovarian tissue^27^.

To initiate immunohistofluorescence (IHF) staining, slides were incubated in antigen retrieval buffer (10 mM Tris base (Sigma-Aldrich), 1 mM EDTA (Sigma-Aldrich), 0.05% Tween 20 (AppliChem, Darmstadt, Germany) in distilled water; pH 9.0) at 90 °C for 20 min. Afterwards, slides were washed in TBST (containing 20 mM Tris base, 150 mM NaCl (Carl Roth, Karlsruhe, Germany)) and 0.05% TritonX-100 (Sigma-Aldrich) in distilled water; pH 7.6). Blocking was conducted due incubation in 5% skim milk powder (Sigma-Aldrich, St. Louis, USA) in TBST for 30 min. The primary antibody against PCNA was diluted in blocking buffer (PCNA: PC10; Cell Signaling Technology, Danvers, MA, USA). Sections were incubated in the solution of primary antibodies overnight at 4 °C in a humidity chamber. Staining controls were incubated without primary antibodies but blocking buffer. After washing in TBST, secondary antibody (goat anti-mouse IgG2a cross-adsorbed secondary antibody; Thermo Fisher Scientific, Waltham, MA, USA) was diluted in blocking buffer and applied for 30 min at room temperature. Slides were washed in TBST and finally rinsed in distilled water. DAPI-containing mounting medium (Carl Roth, Karlsruhe, Germany) was applied. Images were taken with the Eclipse Ti2 microscope (Nikon; Minato, Japan).

### Apoptosis staining (TUNEL)

To investigate cell death as a marker for tissue integrity, terminal deoxynucleotidyl transferase biotin-dUTP nick end labeling (TUNEL) was performed. Two sections of the two tissue samples per treatment group per patient were stained and analyzed.

Tissue sections were stained for DNA double strand breaks (TUNEL BrdU staining kit; abcam, Cambridge, UK) and counterstained for full DNA (DAPI; Carl Roth). TUNEL staining was accomplished following the manufacturer’s description. Briefly, cryoslides were washed in PBS for 5 min twice. For antigen retrieval, the tissue sections were incubated with 20 µg/mL proteinase K (Merck) in Tris–HCl for 5 min at room temperature. After washing in PBS for 5 min, cryosections were incubated in 3.7% formalin in PBS for 5 min and washed in PBS for 5 min. Sections were then covered in Wash Buffer and incubated for 5 min before covered in DNA Labeling Solution. The slides were incubated in the dark at 37 °C for 1 h under humidified circumstances and washed in PBS for 5 min. Then, sections were incubated with the Antibody Solution in the dark for 30 min at room temperature and washed in ddH2O for 5 min twice. Sections were mounted in DAPI-containing mounting medium (Carl Roth). Images were taken using an Eclipse Ti2 microscope (Nikon; Minato, Japan). Sections stained without the usage of the TdT Enzyme were used to assess the background staining/autofluorescence and adjust the settings accordingly. Images were analysed using the Nikon NIS-Elements Software Version 5.30.03 (Nikon; Minato, Japan). Sections were marked as Region of Interest (ROI) using the Auto-tool, which was adapted until the whole section was covered by the ROI (see Fig. 4B). The mean signal intensity of the TUNEL staining inside the ROI was assessed by the NIS-Elements program and exported. Thus, apoptotic changes of all cell types were evaluated. Mean intensity values of the TUNEL staining was assessed. The mean of the two sections of the two tissue samples per treatment group per patient were compared. To ensure comparability of the treatment groups, each slide contained sections of all three groups per patient and were stained simultaneously to avoid inter-experimental variability potentially affecting the results.

## Results

Ovarian tissue from patients undergoing fertility preservation was used in this study. Tissue sections showed the expected characteristics of ovarian cortical tissue with follicles at different stages. Primordial follicles characterized by flat granulosa cells, primary follicles with cuboidal granulosa cells and early secondary follicles with more than a monolayer of cuboidal granulosa cells were observed. Moreover, follicles were viable as shown by the positive staining for PCNA^27, 28^. Preserved viability and integrity of the tissue was observed, as shown in representative images in Fig. 1.

In order to measure the secretion of angiogenic factors, supernatant obtained from the hypoxic in vitro culture of cryopreserved human ovarian tissue was analysed by an angiogenesis array.

Of all ten measured factors, the signal of EGF, HB-EGF and Leptin was below the level of the negative control in the majority of samples, data not shown.

No significant differences were detected comparing angiogenic profiles of vitrified/rapid warmed and slow frozen/thawed samples. Although, the comparison between fresh and former cryopreserved tissue revealed significant differences.

**Figure 3:**
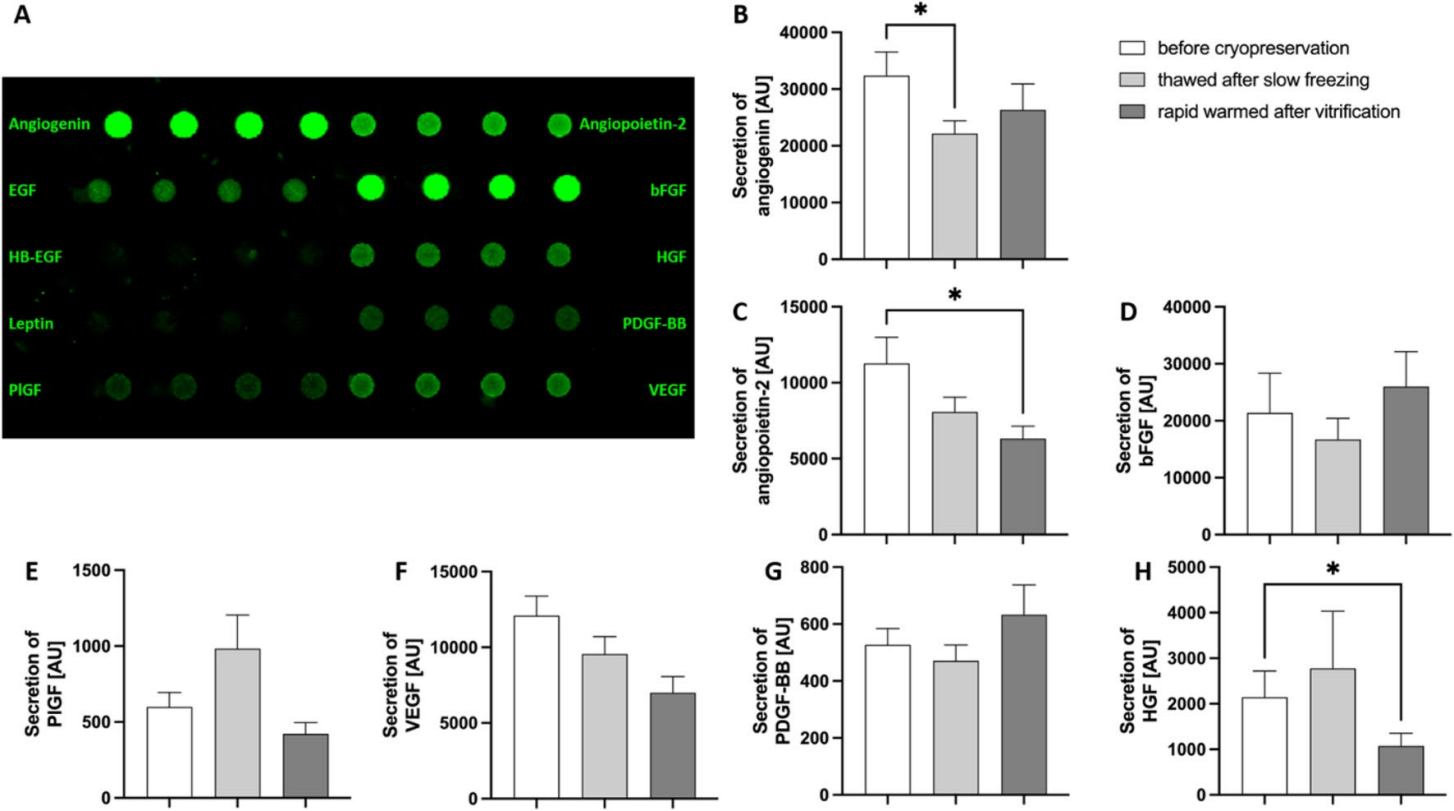
Secretion of angiogenic factors. Supernatant of cultured ovarian tissue was used for angiogenesis array (**A**). Bars show the mean signal intensities of quadruplicates ± SEM (**B**–**H**). Groups were compared by Friedmann test with Dunn’s multiple comparison. n = 20.

Comparing angiogenin levels of slow frozen/thawed samples with fresh samples, we observed a significant decrease (*P* 0.022). Even if decreased, cytokine levels reached at least 50% or more of the corresponding value secreted by fresh tissue, as indicated in Fig. 3 and Table 2.

**Table 2:** Relative secretion [AU] of angiopoietic factors before and after cryopreservation.

Parameter	Fresh min–max SD [AU]	Thawed after slow freezing min–max SD [AU]	Rapid warmed after vitrification min–max SD [AU]		*P value Fresh vs. thawed after slow freezing	*P value Fresh vs. rapid warmed after vitrification	*P value rapid warmed after vitrification vs. thawed after slow freezing
Angiogenin	**32,381.6** 7604.1–95,930.8 18,712	**22,182.7** 9699–41,070 9987.6	**26,360.4** 7099.4–98,825.7 20,337.6	20	**0.0216**	0.3415	0.8051
Angiopoietin 2	**11,274.7** 1884.5–35,523 7676.5	**8072** 859.2–16,122.7 4358.8	**6315.4** 241.3–13,917.8 3665.4		0.3415	**0.0216**	0.8051
bFGF	**21,398.2** 2270.2–145,936.5 31,108.5	**16,714.3** 916–56,890.9 16,613.3	**26,009** 2493.8–87,572.6 27,522.3		0.8051	> 0.9999	0.246
HGF	**2144.7** 515.8–11,262.4 2562	**2775.1** 292.8–25,611 5618	**1076.5** 329–4918.3 1226		0.6177	**0.0342**	0.6177
PDGF-BB	**526.7** 153.1–1153.9 257.3	**471.3** 227.6–1327.8 245.5	**633.3** 225.3–2057.6 466.8		> 0.9999	> 0.9999	> 0.9999
PlGF	**599.3** 112.3–1891.2 426.5	**983.1** 134.7–3768.4 988	**422.4** 120–1456 327.6		0.6177	> 0.9999	0.1195
VEGF-A	**12,090.8** 2882–22,599.9 5762.3	**9560.1** 1369.7–8654.7 5099	**6989.4** 141.8–18,555 4832.5		> 0.9999	0.0806	0.3415

*Friedmann test with Dunn’s multiple comparison.

Significant values are in [bold].

Comparing the levels of angiogenic factors prior to cryopreservation with the release after vitrification/rapid warming, we observed significantly decreased differences with angiopoietin 2 (*P* 0.022) and HGF (*P* 0.034), in addition to a non-significant, decreasing trend for VEGF-A (*P* 0.080). Nonetheless, cytokine measurement has been at a minimum level of 50% or more of the corresponding fresh values, data shown in Table 2 and Fig. 3.

**Figure 4:**
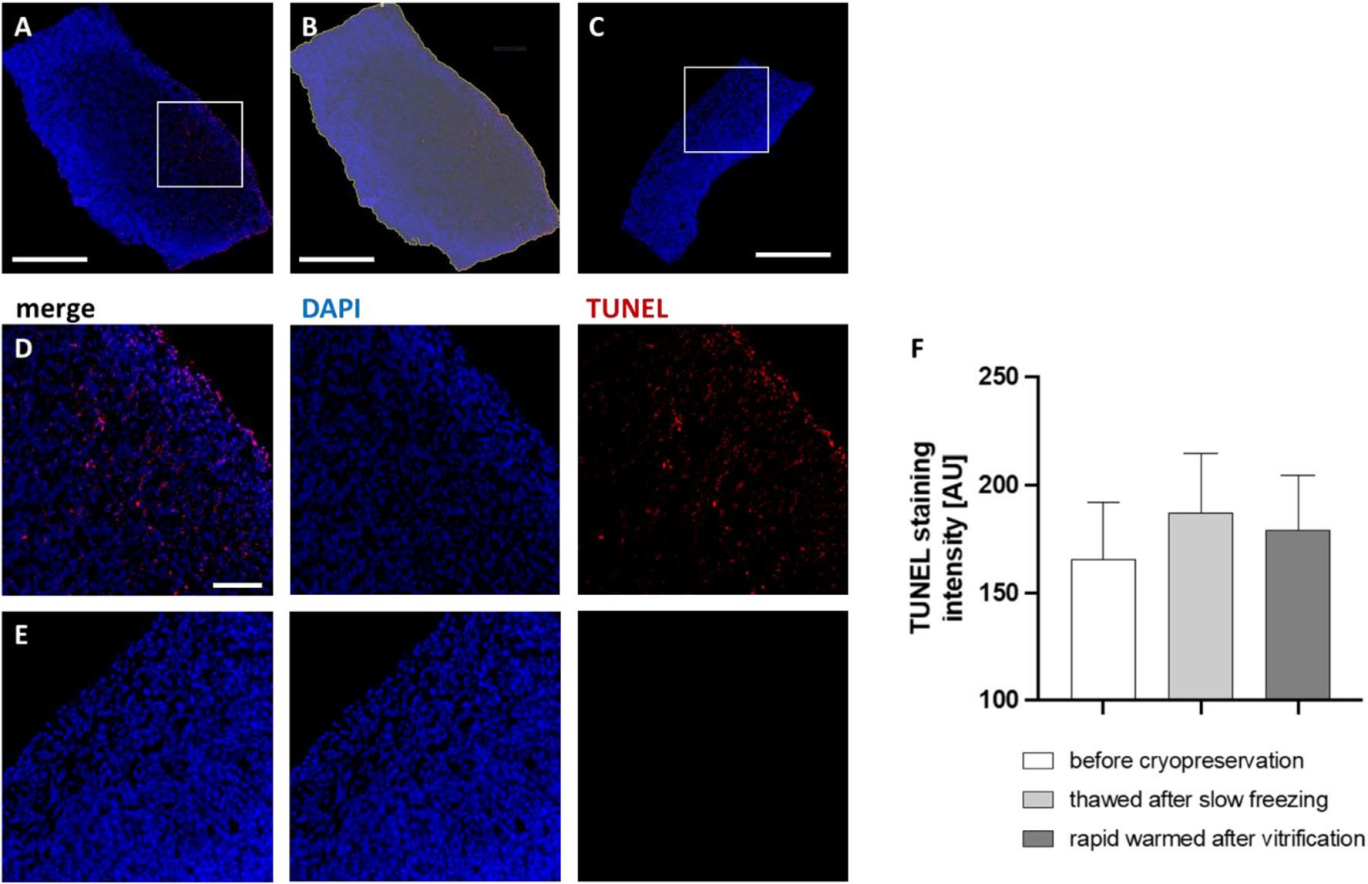
Apoptotic status. Cultured ovarian tissue was used for immunohistofluorescence. TUNEL Assay was applied and counterstained with DAPI. Images of the tissue sections (**A**, **D**) were marked as region of interest (ROI; **B**) and TUNEL signal intensity was assessed. The signal intensity of staining controls (**C**, **E**) was subtracted. Scale bars show 500 µm (**A**–**C**) or 50 µm (**D**, **E**). In the graph, bars show the mean signal intensities of quadruplicates ± SEM (**F**). Groups were compared by Friedmann test with Dunn’s multiple comparison. n = 20.

Our analysis showed low levels of apoptosis. Although there was a tendency to be higher in slow frozen/thawed samples compared to vitrified/rapid warmed samples, no significant differences were found, data shown in Fig. 4.

## Discussion

In this prospective study, we describe the expression pattern of angiogenic factors in supernatants of hypoxic tissue culture of fresh, slow frozen/thawed and vitrified/rapid warmed cortex tissue after 48 h. To estimate the effect of potential cryodamage, tissue sections were stained for nicked DNA (TUNEL).

A proper revascularization is required for the sufficient supply of the tissue, the transfer of critical components is the core function of vascular systems. Due to this, vascularization is pivotal regarding tissue renewal, recovery^29^ and other biological functions^15, 30–35^.

The findings of Rodrigues et al. 2021 implicate that tissue damage due to transplantation may be greater than detrimental effects caused by cryopreservation, as tissue is exposed to post transplantational ischemia until revascularization at the local interface is fully reestablished^36^. In rodents^37, 38^, revascularization is initiated after day 2, while in humans^39^, evidence of reangiogenesis is observed from day 5 after transplantation^40^. Angiogenesis is initiated and mediated by various factors. We showed that, under the chosen culture conditions, human ovarian tissue spontaneously secretes angiogenin, angiopoietin-2, bFGF, HGF, PDGF-BB and VEGF. EGF, HB-EGF and Leptin were barely above the detection limit. The key challenge regarding retransplantation is to counter ischemic deviations^28^, minimized by a prestaggered, optimized cryopreservation/thawing protocol that ensures substantial expression of cytokines involved in angiogenesis as well as a low apoptotic profile of the tissue prior to grafting.

In our study, cytokine expression values after cryopreservation were in general lower than prior to cryopreservation. This is in line with the observation that after cryopreservation the metabolic activity is reduced, accompanied by a vastly decreased secretion of VEGF. This effect recovers over the course of 48 h ^28^. Only few factors were increased after OTC compared to the fresh tissue: bFGF, PDGF-BB (after vitrification/rapid warming), HGF and PlGF (after slow freezing/thawing). Casting a spotlight on these overexpressed factors: bFGF is involved in tissue recovery^41, 42^ and vascularization^43^, besides contributing to protective functions with cerebral ischemia^44–46^. PDGF-BB induces VEGF expression^47^ and angiogenesis^48^. HGF is involved in angiogenesis^49^ and regeneration of tissues^50^ while expression of PLGF mainly occurs in the placenta and contributes to angiopoietic events^51–53^. In the human ovary, bFGF^54^ is involved in folliculogenesis. Whereas in rats, HGF has an anti-apoptotic effect on ovarian granulosa cells^55^. Since PLGF levels in follicular fluid is correlated with ovarian follicle size, it is also speculated to be involved in folliculogenesis^56^. In mice, bFGF also inhibits ischemic oxidative injury in stroke^57^ and myocardial infarct models^58^. After OTC, oxidative stress is the second factor besides hypoxia, which causes a loss of follicles^40, 59^. Described by the term “oxygen paradox”, the reperfusion of ischemic tissue is required for aerobic respiration but causes also a production of reactive oxygen species (ROS), which are highly reactive and able to modify biomolecules up to dysfunctions^59^. Similarly, HGF protects against oxidative stress-induced apoptosis in rat myocardial cells^60^ and PLGF attenuates ROS-mediated injury in a mouse myocardial infarct model^61^. Comparing the cytokine values prior to cryopreservation with samples after thawing/rapid warming, different deviations with single factors were observed while all factors measured reached a minimum level of more than 50% of the cytokine levels of the fresh tissue, potentially contributing to the vascularization capacity after tissue transplantation.

Vascularization is a complex process involving a multitude of single factors potentially triggered by dose-dependent feedback loops in a certain time frame. Due to this, quantitative interpretation of single factors expressed after 48 h remain challenging and must be interpreted cautiously in the overall context.

Studies addressing cryopreservation protocols barely observe the tissue for a prolonged time after thawing or warming. Apoptosis is a programmed type of cell death, which takes hours to days to be completed^62^. Various approaches are available to assess apoptosis. They cover different phases or aspects of the apoptotic cascade and rely on diverse methods. The used TUNEL assay detects nicked DNA, which is a relatively late process in the course of apoptosis. In the course of apopotosis, the stage of double strand breaks cannot be averted in contrast to earlier stages and mark a definite cellular fate^63^. As it takes prolonged time between insult and final cell death, an extended incubation of the tissue is necessary to detect the differences between the cryopreservation protocols properly. Hashimoto et al. let the ovarian tissue of non-human primates equilibrate for 3 h but concluded the interval to be too short^64^. Moreover, Desai et al.^65^ and Xiao et al.^16^ cultured murine and human ovarian tissue for 10 or more days. Both studies showed that differences in outcome and success of the cryopreservation protocols appear after several days, but not immediately after thawing. Vitrification shows improved preservation of the ovarian stromal cells compared to slow freezing^66^. Since stromal cells react more sensitive to stress by the onset of apoptosis than follicles^66–68^, we assessed the onset of apoptosis in the whole ovarian cortical tissue. Our study revealed low apoptotic profiles after 48 h. Although increased after slow freezing/thawing, there was no significant differences between both cryopreservation protocols.

As indicated in our ethical vote, research is constricted to 10% of the tissue, reflecting the limited access of researchers to cryopreserved human ovarian tissue^69^. Due to this, determination of follicular viability via calcein staining was waived as we investigated this question recently with the same cryopreservation protocol detecting no significant differences regarding follicular viability with slow freezing and vitrification^7^.

In summary, we showed that vitrification of human ovarian tissue following our protocol was overall comparable to the results of slow-freezing concerning the release of angiogenic factors and onset of apoptosis. As other advantages of vitrification prevail, such as cost and time effectiveness, we suggest to prefer vitrification/rapid warming over slow freezing/thawing.

## Data Availability

The datasets used and/or analysed during the current study available from the corresponding author on reasonable request.
